# Factor V Leiden mutation triggering four major complications to standard dose cisplatin-chemotherapy for testicular seminoma: a case report

**DOI:** 10.1186/s12894-015-0011-z

**Published:** 2015-03-17

**Authors:** Klaus-Peter Dieckmann, Petra Anheuser, Ralf Gehrckens, Sven Philip Aries, Raphael Ikogho, Wiebke Hollburg

**Affiliations:** Albertinen-Krankenhaus, Department of Urology, Suentelstrasse 11a, D-22457 Hamburg, Germany; Albertinen-Krankenhaus, Department of Diagnostic Radiology, Hamburg, Germany; Elbpneumologie, Mörkenstrasse 47, D-22767 Hamburg, Germany; Hämatologisch-onkologische Praxis Altona (HOPA), Mörkenstrasse 47, D-22767 Hamburg, Germany

**Keywords:** Seminoma, Cisplatin chemotherapy, Thrombosis, Thrombophilia, Factor V Leiden, Bleomycin induced pneumonitis

## Abstract

**Background:**

Major life-threatening complications secondary to cisplatin-based chemotherapy are rare in patients with testicular germ cell tumour (GCT). The incidence of complications increases with dosage of chemotherapy and with a variety of patient-related as well as disease-related conditions. We here report the first case of GCT experiencing as many as four major complications most of which can be explained by the conjunction of several predispositions.

**Case presentation:**

A 48 year old patient with testicular seminoma and bulky retroperitoneal and mediastinal metastases underwent cisplatin based chemotherapy. During the third cycle of chemotherapy, he developed thrombosis of the central venous port device, subtotal splenic infarction, and Bleomycin induced pneumonitis (BIP). Three months after completion of therapy, he was struck by thalamic infarction. Genetic testing then revealed heterozygote mutation of Factor V Leiden (FVL). He received full-dose warfarin anticoagulation treatment and steroid treatment for BIP. 18 months thereafter, the patient is still disease-free, oncologically. Neurological symptoms have disappeared, but pulmonary dysfunction persists with a vital capacity of 50%.

**Conclusion:**

The unique co-incidence of four major complications occurring in this patient were obviously triggered by the genetically determined predisposition of the patient to thrombotic events (FVL). Additionally, several patient-related and disease-related conditions contributed to the unique pattern of complications, i.e. (1) the slightly advanced age (48 years), (2) the prothrombotic condition caused by the disease of cancer, (3) the central venous port device, (4) retroperitoneal bulky metastasis, and (5) cisplatin chemotherapy. Whether or not FVL contributed to the pulmonary fibrosis as well, remains elusive. Practically, in the case of one major vascular complication during cisplatin chemotherapy at standard dose, genetic testing for hereditary thrombophilia should be considered. Thus, precautions for preventing further complications could be initiated.

## Background

Testicular germ cell tumours (GCTs) represent a paradigm for a curable neoplasm [1]. Combination chemotherapy with cisplatin, etoposide and bleomycin (PEB) can cure metastasized disease and even far advanced disease (i.e. poor prognosis according to the IGCCCG classification) can be salvaged in about 50% [2]. However, systemic treatment is quite toxic even by application of standard doses and it involves the potential of numerous adverse reactions arising acutely at the time of treatment and other sequels developing in the long term [3]. The most frequent untoward reactions occurring at the time of treatment or immediately thereafter encompass myelodepression with neutropenic fever, alopecia, gastrointestinal toxicity, peripheral neurotoxicity, and impairment of fertility. These side-effects are experienced by almost all of the patients with varying degrees of severity, and usually, these events can be managed successfully. However occasionally, more serious and even life threatening complications may ensue mainly involving vascular and pulmonary events. Deep venous thrombosis (DVT) is encountered in about 8% of GCT patients receiving cisplatin-based chemotherapy for metastatic disease [4]. Pulmonary embolism secondary to DVT may occur in up to 6% of cases [5,6]. Arterial complications e. g. peripheral arterial thrombotic occlusion, myocardial infarctions, and cerebral vascular events are reported in about 0.3% of GCT patients receiving chemotherapy [7]. Pulmonary fibrosis represents the most important complication involving the lungs. This complication is also called bleomycin induced pneumonitis (BIP) because it is clearly associated with bleomycin if given in cumulative dosages of more than 300 mg [8,9]. About 7% of chemotherapy cases will experience BIP with 1% being fatal [10].

Multiple complications occurring synchronously or metachronously in one patient have been reported [4,7]. But rarely, more than three serious complications will be encountered in one individual [11]. Here we report a case who experienced as many as four serious complications secondary to cisplatin-combination chemotherapy, three vascular events and one pulmonary.

## Case presentation

**Figure 1:**
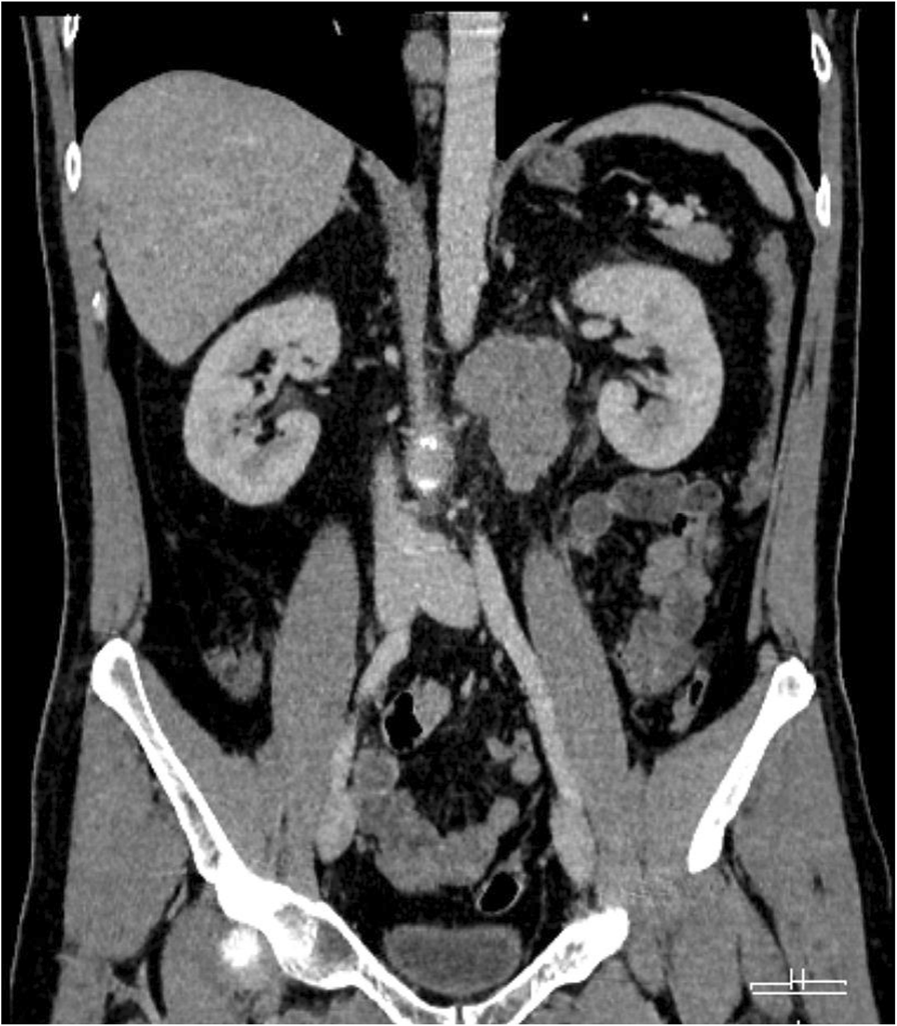
**Abdominal computed tomography, coronary section view showing the large retroperitoneal metastasis of seminoma before initiation of chemotherapy.**

This 48 years old patient with uneventful history presented with a left sided testicular seminoma. Beta Human chorionic gonadotropin (bHCG) was elevated to 247 U/l (normal range to 2 U/l) while Alpha fetoprotein and Lactate dehydrogenase were within normal limits. Computed tomography of chest and abdomen revealed a retroperitoneal mass of 6 cm in size (Figure 1) and another one of 2 cm size in the mediastinum corresponding to clinical stage III (Lugano Classification) and to the good prognosis group according to IGCCCG classification [12].

**Figure 2:**
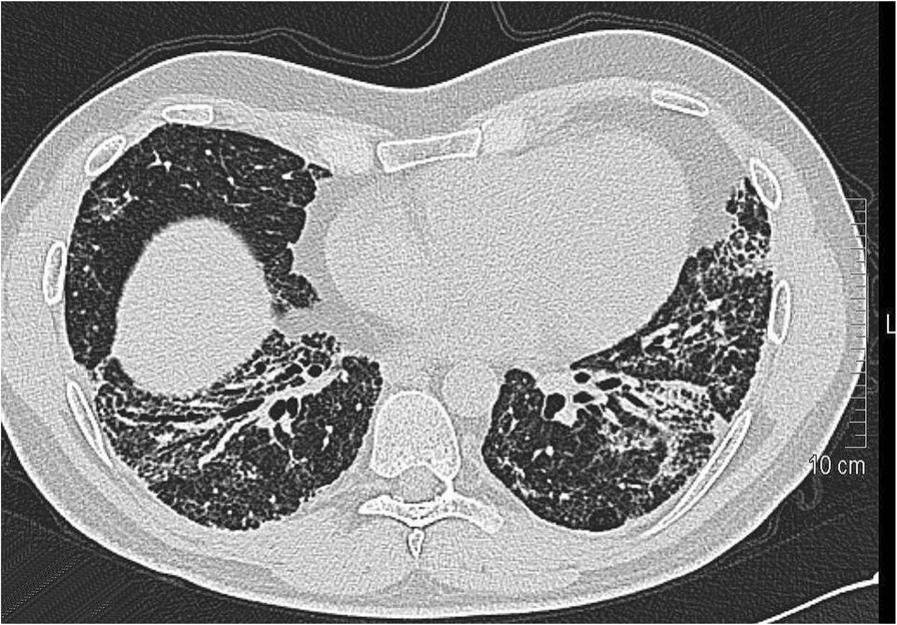
**Chest Computed tomography performed after third cycle of chemotherapy showing diffuse interstitial infiltrates, bilaterally, consistent with Bleomycin induced pneumonitis.**

**Figure 3:**
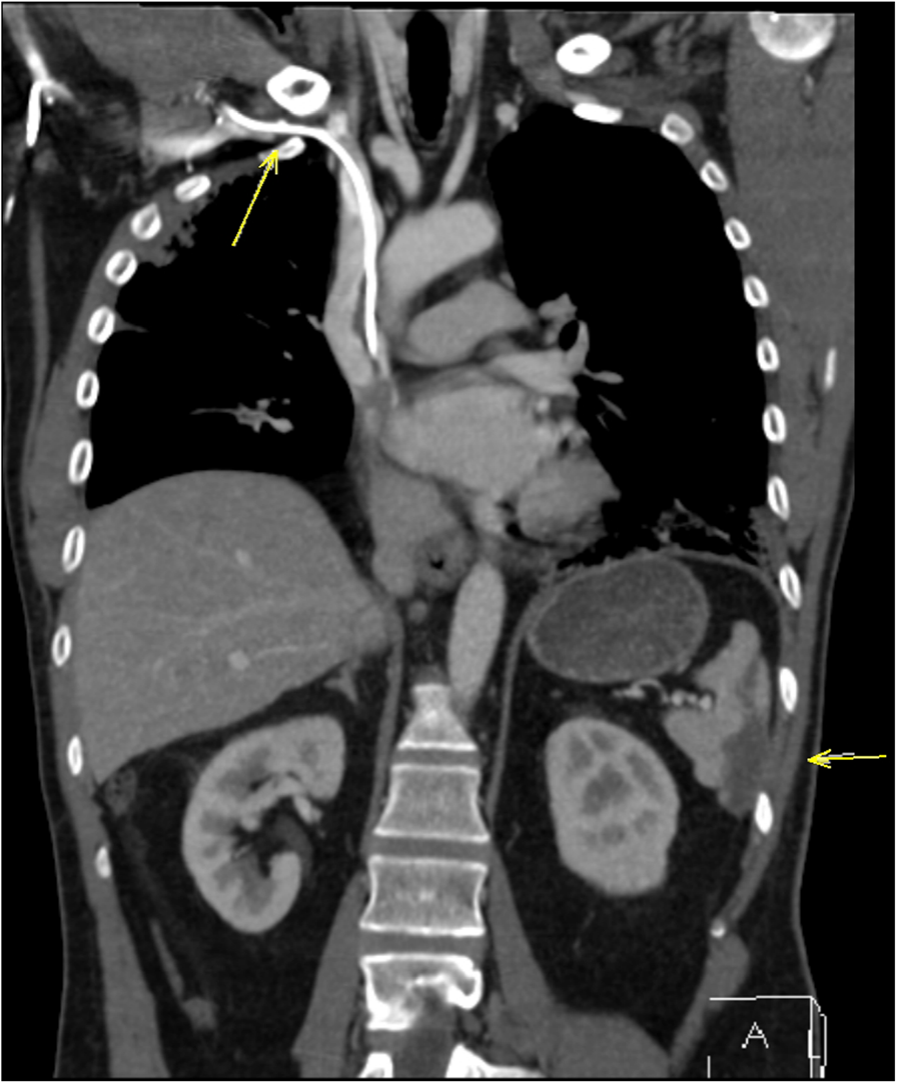
**Computed tomography of chest and abdomen, coronary section, performed after third cycle of chemotherapy, showing the central venous port device with thrombosis (arrow) and subtotal splenic infarction (arrow).**

**Figure 4:**
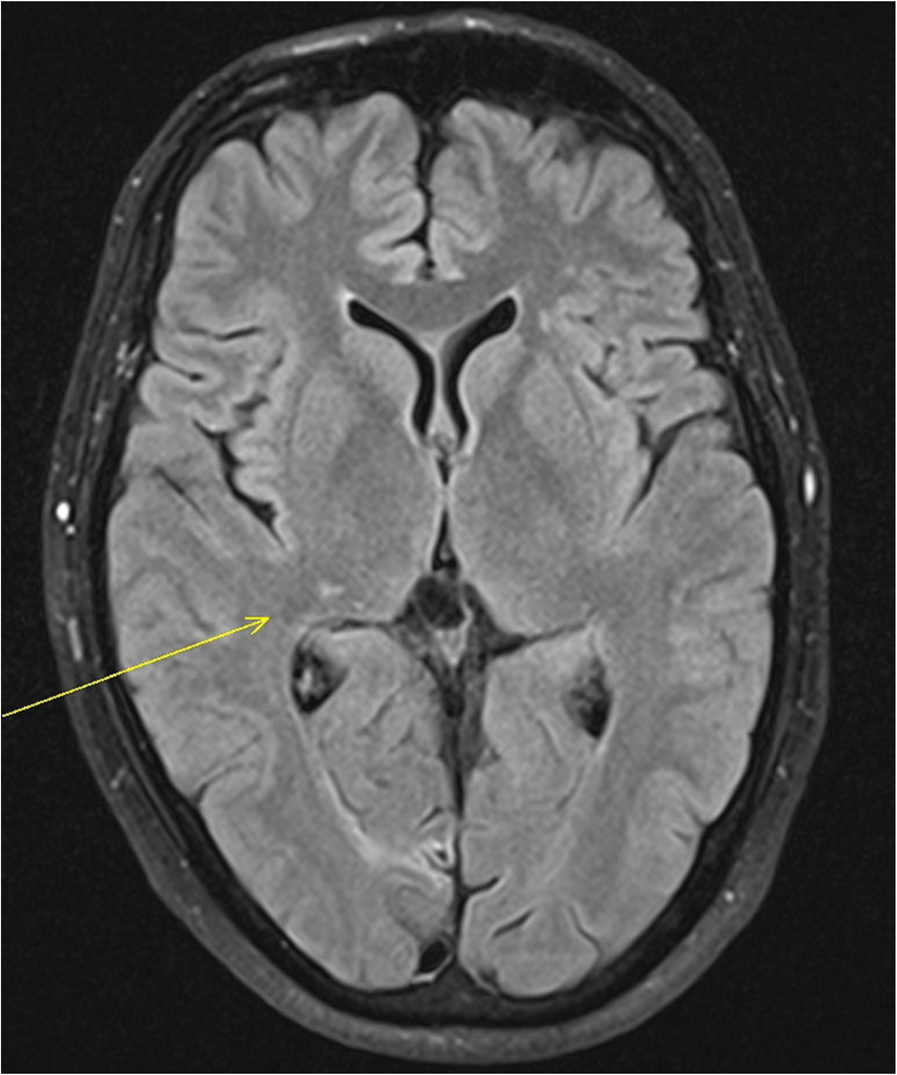
**Axial Magnetic resonance Imaging (MRI) of the Brain with Fluid Attenuated Inversion Recovery (FLAIR sequence), three months after completion of chemotherapy: Hyperintense lesion in the right thalamic region (arrow) indicating thalamic infarction.**

The patient underwent inguinal orchiectomy and according to the patient´s wish, a centralvenous port system with access to the right subclavian vein was implanted at the same time. Low-dose anticoagulation treatment was instituted thereafter with low molecular weight heparin (Enoxiparin 40 mg daily). Three cycles of the cisplatin/etoposide/ bleomycin chemotherapy regimen (PEB) were administered without delay between the cycles. Acute side effects involved recurrent singultus during all cycles, complete alopecia after the third cycle. Routine laboratory examinations done weekly during chemotherapy revealed transient increase of gamma glutarate dehydrogenase, as well anemia with hemoglobin of 8.9 g/dl. Serum creatinine increased during the application of the third cycle of treatment to 1.5 mg/dl indicating a considerable impairment of renal function. Also during the third cycle, the patient reported of increasing dyspnea upon slight exercise. In addition, at the end of the third cycle, the patient experienced a sudden episode of left upper abdominal pain. Routine restaging with computed tomography of chest and abdomen after completion of the second cycle of chemotherapy revealed partial remission of the lymphadenopathy but no other significant findings. Final restaging after the third cycle documented complete remission of the mediastinal mass and subtotal remission of the retroperitoneal mass. Accordingly, serum bHCG had returned to normal. Thus, cure had been achieved, oncologically. However, chest CT also revealed fibrotic changes bilaterally in the caudal parts of the lungs consistent with the typical findings in Bleomycin induced pneumonitis (Figure 2). Accordingly, pulmonary function testing revealed a decrease of vital capacity to 44%. Also, the chest CT revealed thrombosis of the centralvenous port system with extension of thrombotic material into the subclavian vein (Figure 3). In addition, the abdominal CT revealed splenic infarction involving approximately one third of the organ (Figure 3). Therapeutically, anticoagulation therapy was increased to 80 mg Enoxaparin daily and the venous port system was removed surgically. In addition, corticosteroid therapy with 50 mg Prednisolone p/o was applied for treatment of BIP resulting in some improvement of dyspnea, subsequently. Three months after completion of chemotherapy and despite ongoing full dose heparin-based anticoagulation therapy, the patient experienced a sudden loss of speech and amnesic aphasia. Magnetic resonance imaging of the brain disclosed infarction of the right-sided thalamic region (Figure 4) as well as several additional small older ischemic regions in the temporal and occipital cortex, respectively.

After all these complications genetic testing for inherited thrombophilia documented heterozygote mutation of factor V Leiden (FVL).

The patient was placed on life-long warfarin anticoagulation therapy. 18 months after completion of chemotherapy, follow-up examinations revealed ongoing complete remission, oncologically. The neurological symptoms had resolved totally. The corticosteroid therapy had been terminated 14 months after onset of BIP. Exertional dyspnea has improved meanwhile, however, vital capacity is still no more than 50%.

## Conclusion

Generally, the occurrence of complications secondary to a chemotherapeutic agent is dependent on both the applied dosage of the drug and the predisposition of the individual patient to acquire particular complications. Accordingly, high dose chemotherapy is associated with a much higher frequency of major complications. With respect to the susceptibility of the patient to acquire complications, older age is clearly one general predisposing factor as well as pre-existing chronic diseases e.g. Diabetes mellitus, renal insufficiency, chronic vascular disease. As most of the patients with testicular GCT are young and otherwise healthy, and as most of the metastasized cases only require standard dose chemotherapy, major complications are rather infrequent. Two or more critical events arising simultaneously or consecutively in one such patient are even more infrequent. Thus, the conjunction of four serious events as experienced by our patient is truly exceptional. O´Reilly reported a 26 year old patient with testicular seminoma and no significant comorbidity who sequentially experienced cerebral stroke, deep vein thrombosis and pulmonary embolism during PVB chemotherapy [11]. A 33 year old Dutch patient with GCT who was otherwise healthy developed cerebral stroke and myocardial infarction upon cisplatin-based therapy [13]. Likewise, in a German series, two patients (36 and 44 years of age) with both, myocardial infarction and cerebral stroke were reported. Also in that report, five cases with multivessel myocardial infarction as well as two cases with pulmonary embolism occurring in conjunction with cerebral stroke and with peripheral arterial thrombosis, respectively, were documented [7].

With respect to the present patient, it is probably the unique combination of a number of particular conditions that instigated this extraordinary pattern of untoward events.

Undoubtedly, the thrombophilic predisposition of this patient documented by the mutation of Factor V Leiden (FVL) represents the paramount pathogenetic factor that triggered the major vascular complications. Heterozygote status of FVL mutation is prevalent in around 5% of the Caucasian population involving a seven-fold increase of the thrombogenic risk in afflicted subjects [14]. Evidently, FVL does also modestly increase the risk of arterial thrombosis [15-17], at least in the presence of additional prothrombotic conditions [18]. Even splenic infarction, one of the events of our patient, has been documented in a case with FVL mutation [19].

Thus, our patient was bearing a genetically determined significantly increased risk of thrombosis which was superimposed by at least four additional synergistic thrombogenic factors: age, cancer, central venous port system, and cisplatin chemotherapy. (I) Firstly, the slightly advanced age of 48 years confers a minor increase of general thrombotic risk by comparison to the average GCT patient. (II) Cancer itself generally increases the risk of thrombosis [20,21]. Moreover, there is also clear evidence of further increased thrombotic risk in those individuals who have both, cancer and FVL mutation [14]. On top of that, our patient had a huge retroperitoneal mass that potentially might compromise venous backflow from the lower limbs thus predisposing to intravasal clotting [4]. (III) Central venous port devices generally involve a high potential of thrombosis due to contact and irritation of vascular walls with the synthetic port material [22]. In the presence of FVL mutation, an additional 3 – 6 fold thrombotic risk has been documented [23,24]. (IV) Cisplatin combination chemotherapy involves considerable thrombogenic risk [25] possibly caused by endothelial damage [6,26,27]. Whether or not anti-emetic steroid therapy and BIP-related corticoid administration may have advanced the thrombotic risk in our patient is at least conceivable [28]. In all, our patient experienced three thrombotic events during or immediately after cisplatin-based chemotherapy. Obviously these complications were initiated and promoted by the conjunction of the patient´s genetically determined increased risk of thrombosis on the one side, and several disease-related as well as treatment related synergistic prothrombotic factors on the other side.

With regard to the Bleomycin-related pulmonary fibrosis the patient used to be a non-smoker and he had no apparent predisposing pulmonary conditions. Yet, two minor precipitating factors could be noted, i.e. the slightly advanced age of 48 years and the impaired renal function developing during the third cycle of chemotherapy, both of these conditions are known to be associated with the occurrence of BIP [8]. At least on the experimental level there is some evidence that FVL mutation may promote pulmonary fibrosis, too [29]. Thus conceivably, the pulmonary and the vascular complications occurring in the present patient may share predisposing factors.

From a clinical point of view, it must be asked how and if at all these events could have been prevented. Probably, the pulmonary complication represents much of a chance event that had not been predictable at the time of treatment. Two of the three vascular complications were endo-arterial events. So, low dose heparin anticoagulation would probably not have been sufficient to prevent further vascular events. If acetylic salicylic acid (ASA) would have been beneficial in this case remains elusive. However, what should be learned from this extraordinary case is that genetic testing for hereditary clotting disorders should be considered in all those GCT patients experiencing one major vascular complication. Then, life-threatening further complications could be prevented by full dose warfarin medication or other modern oral anticoagulation treatment.

## Consent

Written informed consent was obtained from the patient for publication of this Case report and the accompanying images. A copy of the written consent is available for review by the Editor of this journal.

## Abbreviations

GCT: Germ cell tumour
PEB: Cisplatin, Etoposide, Bleomycin (chemotherapy)
IGCCCG: International germ cell cancer consensus group
BIP: Bleomycin induced peumonitis
CT: Computed tomography
FVL: Factor V leiden
DVT: Deep vein thrombosis
BHCG: Beta human chorionic gonadotropin
p/o: Per oral (medication)
